# A Rare Large Colonic Xanthoma Mimicking Neoplasia in a Young Adult: An Unusual Finding During an Iron Deficiency Anemia Workup

**DOI:** 10.7759/cureus.88433

**Published:** 2025-07-21

**Authors:** Abdulrahman A Almalaq, Abdullah M Albishi, Majed S Alzahrani, Muneerah A Alzouman, Abdulrhaman A Alrobayan

**Affiliations:** 1 Gastroenterology and Hepatology, Prince Sultan Military Medical City, Riyadh, SAU; 2 Internal Medicine, Prince Sultan Military Medical City, Riyadh, SAU; 3 Histopathology, Prince Sultan Military Medical City, Riyadh, SAU

**Keywords:** anemia, endoscopic mucosal resection, polyps, xanthoma, colonoscopy

## Abstract

Colorectal xanthomas are rare, benign lesions characterized by collections of foamy histiocytes within the lamina propria. Although commonly seen in the stomach, their occurrence in the colon is exceedingly uncommon and can mimic neoplastic or serrated lesions endoscopically. We report the incidental discovery of a large colorectal xanthomatous polyp in a 37-year-old male undergoing colonoscopy for the evaluation of persistent iron deficiency anemia. A sessile, pale-white lesion measuring 20 mm × 20 mm with a smooth surface and central depression was identified in the descending colon and resected via endoscopic mucosal resection (EMR). Histopathologic examination revealed foamy histiocytes positive for CD68 and negative for S100, consistent with a xanthoma. This case highlights the importance of considering xanthoma in the differential diagnosis of atypical-appearing colonic polyps and underscores the need for histologic confirmation to avoid overtreatment.

## Introduction

Xanthomas are benign, non-neoplastic lesions characterized by the accumulation of lipid-laden foamy histiocytes within the lamina propria of mucosal tissues. While commonly found in the stomach, esophagus, and skin, their presence in the colon is exceedingly rare, with most cases discovered incidentally during routine colonoscopy or workup for nonspecific gastrointestinal symptoms [1-3].

The pathogenesis of colorectal xanthomas remains incompletely understood, but proposed mechanisms include chronic mucosal injury, altered lipid metabolism, local inflammation, and tissue repair following epithelial damage [4-6]. They have been observed more frequently in elderly patients and may occasionally be associated with conditions like hyperlipidemia or prior gastrointestinal surgery [7-9].

Endoscopically, colonic xanthomas typically appear as small, pale, flat, or slightly elevated lesions with a smooth surface and absent vascular markings, often mimicking sessile serrated lesions or superficial neoplasia [10]. Their unusual appearance can lead to diagnostic confusion and often necessitates histopathologic confirmation. On microscopy, they are defined by aggregates of foamy histiocytes positive for CD68 and negative for S100, helping to exclude mimics such as lipomas, mucosal prolapse, and signet-ring cell carcinoma [11-13].

Although generally asymptomatic and clinically insignificant, colorectal xanthomas have occasionally been reported in association with polyps or adenomas, raising questions about their biological relevance or possible reactive nature [14,15].

Here, we present a case of an unusually large, flat colorectal xanthomatous lesion identified during the evaluation of iron deficiency anemia in a young adult male. The lesion was resected via endoscopic mucosal resection (EMR) due to its atypical morphology and potential for neoplastic mimicry. This case adds to the limited but growing literature on this rare entity and highlights the importance of histological evaluation in distinguishing benign from potentially malignant colonic lesions.

## Case presentation

A 37-year-old male with no significant past medical history aside from a sleeve gastrectomy in 2019 was referred for the evaluation of persistent iron deficiency anemia (IDA). He was asymptomatic aside from fatigue, with normal vital signs and unremarkable physical examination.

Initial laboratory tests showed results were consistent with iron deficiency anemia and mildly elevated low-density lipoprotein (LDL). Tumor markers, including carcinoembryonic antigen (CEA), were within normal limits. Celiac serology, renal, liver, and thyroid function tests were within normal limits.

An additional anemia workup, including fecal occult blood testing, was negative, and celiac serology. Tissue transglutaminase immunoglobulin (IgA) was negative, and total serum IgA was within normal limits, supporting the exclusion of celiac disease. Vitamin B12 and folate levels were within normal limits. There was no evidence of hemolysis on peripheral smear or biochemical markers (Table 1).

**Table 1:** Laboratory findings at presentation

Test	Result	Reference Range	Unit
Hemoglobin	106	130–170	g/L
Hematocrit	0.347	0.40–0.50	Ratio
Mean Corpuscular Volume (MCV)	70	80–100	fL
Ferritin	7	30–400	µg/L
Iron	5	10–30	µmol/L
Total Iron-Binding Capacity (TIBC)	66	45–72	µmol/L
Low-Density Lipoprotein (LDL) Cholesterol	3.1	< 3.00	mmol/L
Carcinoembryonic Antigen (CEA)	2	< 3.0	µg/L
Vitamin B12	387	140–700	pmol/L
Folate	13	> 7	nmol/L
Tissue Transglutaminase Immunoglobulin A (tTG-IgA)	Negative	Negative	–
Total IgA	1.9	0.70–4.00	g/L
Peripheral Smear and Hemolysis Markers	Normal morphology and markers		

**Figure 1:**
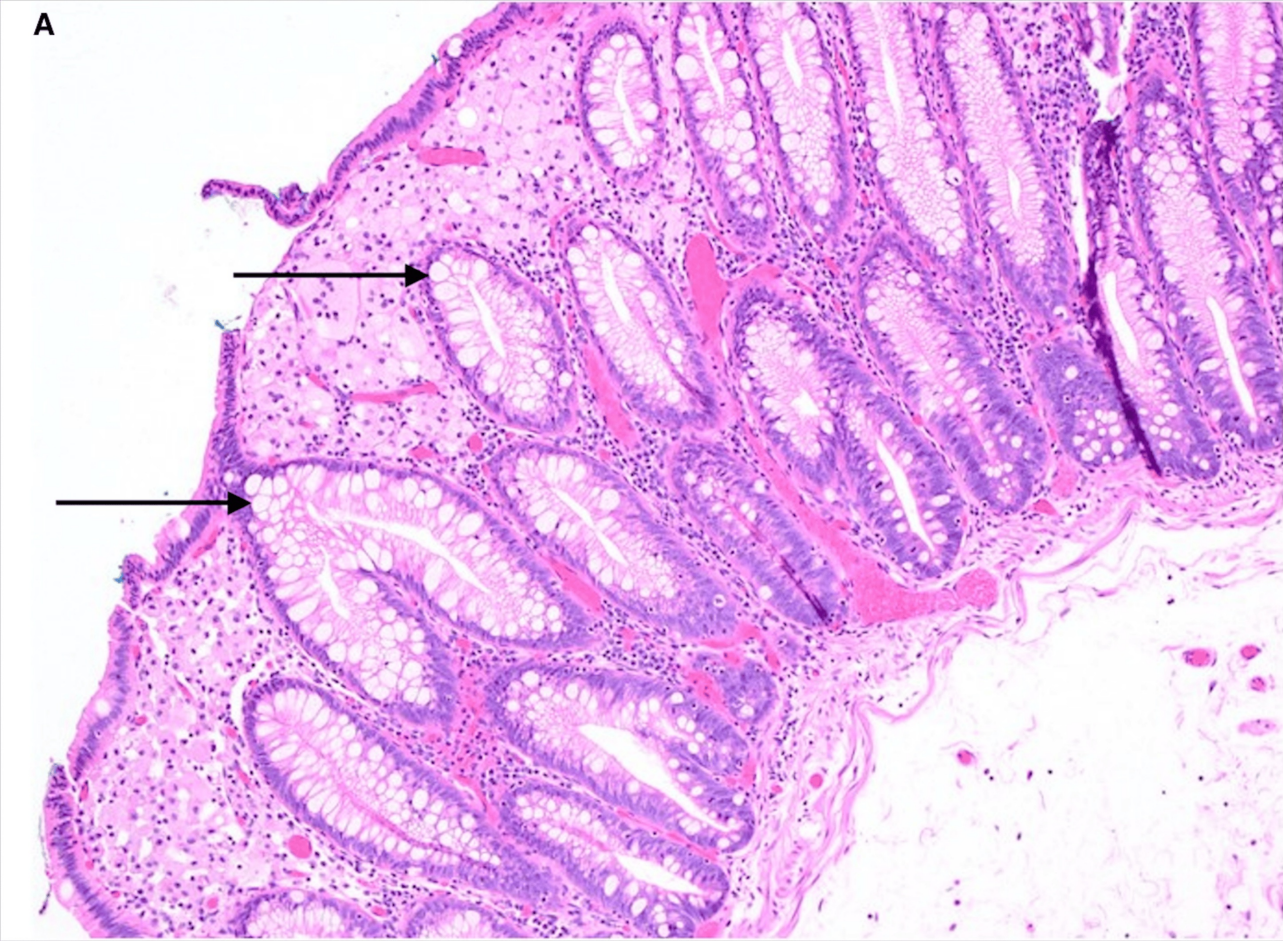
Histologic section of the resected lesion (A)Histologic section of the resected lesion showing clusters of foamy histiocytes in the lamina propria (H&E stain, original magnification ×100)

Although IDA is a known complication after sleeve gastrectomy, the persistence of anemia despite oral iron supplementation and the patient’s male sex prompted full endoscopic evaluation. Gastroduodenoscopy was unremarkable. Colonoscopy revealed excellent bowel preparation (Boston Bowel Preparation scale: 9/9). A solitary, flat, pale-white elevated lesion measuring approximately 20 mm × 20 mm was identified in the descending colon. The lesion exhibited a slightly granular surface and central depression, with absent vascular markings on narrow-band imaging (NBI). The lesion met criteria for Paris Type 0-IIa, indicating a slightly elevated superficial morphology. These atypical features, including the flat morphology, color, and absence of normal vascular pattern, raised concern for a sessile serrated lesion or superficial neoplasia, justifying resection via EMR. Histopathologic analysis showed clusters of foamy histiocytes within the lamina propria without dysplasia (Figure 1A). Immunohistochemical staining was strongly positive for CD68 (Figure 1B), confirming the diagnosis of colorectal xanthoma. The histologic appearance ruled out mimics such as mucosal prolapse, lipoma, and signet-ring cell carcinoma.

**Figure 2:**
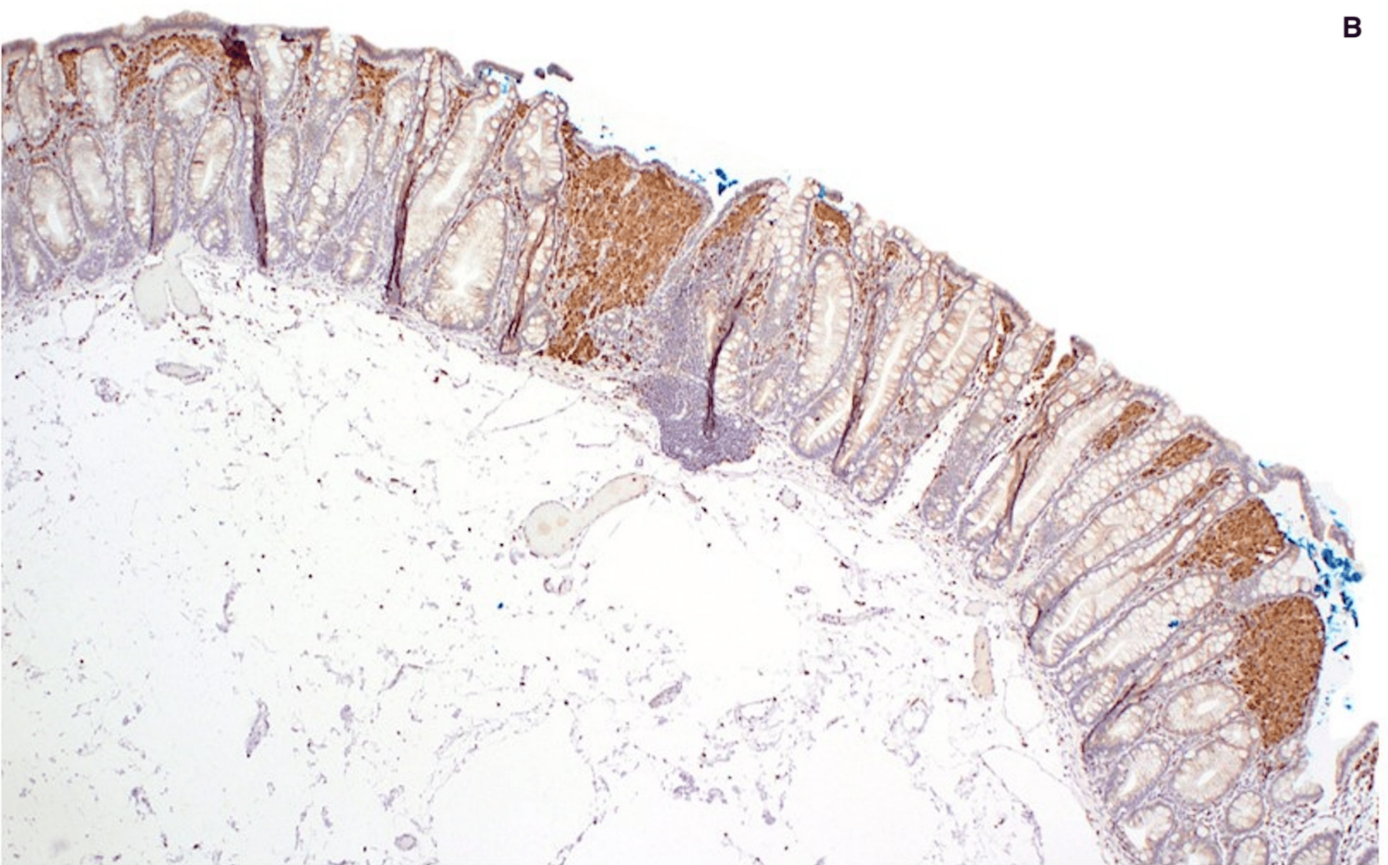
Immunohistochemical staining of the resected lesion (B) Immunohistochemical staining was strongly positive for CD68, confirming the diagnosis of colorectal xanthoma.

The patient continued oral iron therapy post-polypectomy, and follow-up revealed gradual improvement in hemoglobin and ferritin levels. While causality cannot be confirmed, the hematologic improvement raises the possibility that the lesion may have contributed to chronic occult blood loss below the detection threshold of the screening test.

## Discussion

Colorectal xanthomas are rare, benign, non-neoplastic lesions characterized by the accumulation of lipid-laden foamy histiocytes within the lamina propria. While gastric and esophageal xanthomas are more frequently encountered, colonic involvement is exceptionally uncommon, with fewer than 50 cases described in the English literature to date [1-6]. Most reported cases are incidental findings during routine screening colonoscopy, usually presenting as small, flat, or sessile polyps, often less than 10 mm in size [2,4,5].

Our case is unique for several reasons. First, the lesion was notably large, measuring 20 × 20 mm, placing it among the largest colonic xanthomas reported in the literature. Nakasono et al. analyzed 25 cases of polypoid colorectal xanthomas and found that most were smaller than 10 mm, with only a minority exceeding 15 mm [2]. Similarly, Miliauskas reported 4 rectosigmoid cases ranging from 3 to 12 mm in diameter [1]. In contrast, our case’s size raised concern for neoplasia and warranted resection by endoscopic mucosal resection (EMR), emphasizing its clinical significance.

Second, the lesion’s endoscopic appearance was atypical and potentially misleading. It presented as a flat (Paris Type 0-IIa), pale-white lesion with a central depression and absent vascular markings-features more suggestive of a sessile serrated lesion or early neoplasia than a benign xanthoma. Prior case reports have described colonic xanthomas as yellowish or pale nodules but rarely emphasized this particular morphology [4,6,8]. This underlines the diagnostic challenge posed by such lesions and supports the necessity of histologic confirmation, especially when visual features overlap with dysplastic or malignant polyps.

Third, our patient was relatively young (37 years old) and had a prior history of sleeve gastrectomy. While no direct causative relationship has been established, it is plausible that altered gastrointestinal physiology or mucosal remodeling post-bariatric surgery may play a role in xanthoma formation, possibly through chronic mucosal irritation or lipid malabsorption. Few reports have linked GI xanthomas to metabolic derangements, such as hyperlipidemia [4,9], and even fewer have discussed prior gastrointestinal surgeries as contributing factors.

Notably, recent literature has highlighted rare associations between xanthomas and neoplastic changes. For instance, Jung et al. reported a case of an adenoma arising within a transverse colon xanthoma [7], and Coffey et al. described a tubular adenoma overlying a rectosigmoid xanthoma [6]. While our case showed no dysplasia, the neoplastic mimicry further justifies excision and close histological evaluation.

Histologically, xanthomas consist of aggregates of foamy histiocytes that are strongly positive for CD68 and negative for S100 protein, as seen in our case. This immunoprofile helps distinguish them from mimics such as lipomas, mucosal prolapse, signet-ring cell carcinoma, and other conditions positive for both CD68 and S100, like Langerhans cell histiocytosis and dendritic cell tumor [4,10-12]. The absence of atypia, mitoses, or architectural distortion confirmed the benign nature of the lesion.

## Conclusions

This case contributes to the limited but growing body of literature on colorectal xanthomas, particularly in younger patients and in the context of iron deficiency anemia. It also reinforces that lesions with atypical endoscopic features, even when benign, should be resected for a definitive diagnosis, especially when found during an anemia workup.
